# Gut microbial dysbiosis is associated with development and progression of radiation enteritis during pelvic radiotherapy

**DOI:** 10.1111/jcmm.14289

**Published:** 2019-03-25

**Authors:** Zhongqiu Wang, Qingxin Wang, Xia Wang, Li Zhu, Jie Chen, Bailin Zhang, Ye Chen, Zhiyong Yuan

**Affiliations:** ^1^ Department of Radiation Oncology, Tianjin Medical University Cancer Institute and Hospital, National Clinical Research Center for Cancer Tianjin's Clinical Research Center for Cancer, Key Laboratory of Cancer Prevention and Therapy Tianjin China; ^2^ Department of Biomedical Engineering Tianjin University Tianjin China; ^3^ Department of Gastrointestinal Oncology, Tianjin Medical University Cancer Institute and Hospital, National Clinical Research Center for Cancer Tianjin's Clinical Research Center for Cancer, Key Laboratory of Cancer Prevention and Therapy Tianjin China; ^4^ Department of Gastroenterology, Guangdong Provincial Key Laboratory of Gastroenterology Nanfang Hospital, Southern Medical University Guangzhou China

**Keywords:** 16S rRNA gene sequencing, cervical cancer, gut microbiota, radiation enteritis, radiotherapy

## Abstract

Radiation enteritis (RE) is the most common complication of radiotherapy for pelvic irradiation receivers. Herein we investigated the alterations in gut microbial profiles and their association with enteritis in patients undergoing pelvic radiotherapy. Faecal samples were collected from 18 cervical cancer patients during radiotherapy. Microbiota profiles were characterized based on 16S rRNA sequencing using the Illumina HiSeq platform. Epithelial inflammatory response was evaluated using bacterial‐epithelial co‐cultures. Dysbiosis was observed among patients with RE, which was characterized by significantly reduced α‐diversity but increased β‐diversity, relative higher abundance of *Proteobacteria* and *Gammaproteobacteria *and lower abundance of *Bacteroides* *.* *Coprococcus *was clearly enriched prior to radiotherapy in patients who later developed RE. Metastat analysis further revealed unique grade‐related microbial features, such as more abundant *Virgibacillus* and *Alcanivorax *in patients with mild enteritis. Additionally, using bacterial‐epithelial co‐cultures, RE patient‐derived microbiota induced epithelial inflammation and barrier dysfunction, enhanced TNF‐α and IL‐1β expression compared with control microbiota. Taken together, we define the overall picture of gut microbiota in patients with RE. Our results suggest that dysbiosis of gut microbiota may contribute to development and progression of RE. Gut microbiota can offer a set of biomarkers for prediction, disease activity evaluation and treatment selection in RE.

## 1 INTRODUCTION

The widespread use of radiation has led to high incidence of radiation‐induced side effects in up to 75% of radiotherapy receivers.^1^ Among pelvic radiotherapy receivers, approximately 90% of them develop a permanent change in their bowel habit after irradiation, and 50% have an associated reduction in quality of life.^2^ Although considerable progress has been made to reduce bowel toxicity of radiotherapy, the most commonly adopted approach now is still reduction of the delivered radiation dose, which may inevitably decrease treatment efficacy.^3^, ^4^ There is neither standardized prophylactic nor therapeutic strategies available proven to mitigate the radiation enteritis (RE) symptoms or allow safe radiation dose escalation for better cancer control. In 2002, Pietro Delia^5^ reported that use of *Lactobacillus rhamnosus *could prevent the occurrence of diarrhoea in patients receiving radiotherapy. Later, Crawford and Gordon^6^ revealed that germ‐free mice were markedly resistant to lethal RE. And after faecal microbiota transplantation, the intestinal function and survival rate were significantly improved in irradiated mice.^7^ These reports suggest a human‐microbiome link upon irradiation and provided an insight into potential radio‐protective therapeutics.

The advancement of recent next‐generation sequencing technologies and bioinformatics has changed the way research is done in microbial ecology. It analyses the complete bacterial genome sequences and provides enormous amounts of information.^8^ Direct evidence of alterations in the overall composition of the gastrointestinal microbiome has been reported in various studies. Dinakaran et al^9^ identified different patterns of colon microbiome between Caucasians and African‐Americans. They also found that the proportion of bacteria in the inflammatory bowel disease samples was altered compared to adjacent healthy samples. Lavelle et al^10^ demonstrated spatial variation between the luminal and mucosal microbiome in ulcerative colitis and healthy controls. In addition, Nan et al^11^ revealed 75 245 genes differing between liver cirrhosis patients and healthy individuals, and most of the patient‐enriched species were of buccal origin, suggesting an invasion of the gut from the mouth.

In this study, using the high‐throughput 16S rRNA gene sequencing, we identified specific faecal microbial signatures in patients with RE and sought to elucidate potential biomarkers or mechanistic principles how the gut microbiota dysbiosis may impact the pathogenesis of RE. The results will also provide useful information about the therapeutic value of microecological preparation for RE.

## 2 MATERIALS AND METHODS

### 2.1 Sample collection

Eighteen patients with stage II‐IV cervical cancer (CCa) who had not received any treatments for those conditions and were undergoing pelvic radiotherapy were recruited in our department from June 2015 to January 2016. The detailed clinical parameters are shown in Table 1. The exclusion criteria were as follows: recent (<2 months prior) use of any antibiotic or probiotic therapy, recent (<2 weeks prior) use of any proton pump inhibitors, known any other enteritis,^9^, ^10^ known autoimmune condition, significant gastrointestinal disorder, age <18 years, vegetarians, abnormal BMI value (<18.5 or >24), known history of any other cancer,^12^ and significant liver, renal, or peptic ulcer disease. Baselines of bowel habit and symptom were recorded, and patients with prior higher bowel symptoms were excluded as well. Pelvic radiotherapy was delivered at total doses of 50.4 Gy in 1.8 Gy/fraction. Diagnoses of RE depended on the combination of clinical symptoms (eg abdominal pain, tenesmus, rectal bleeding, faecal incontinence, diarrhoea or vomiting without other obstructive symptoms), medical histories and exclusion of other potential diagnoses. Faecal samples were obtained one day before and at the first day after the treatment. Approximately 5 g of faecal samples were collected and immediately frozen at −20°C, then stored at −80°C until further processing. Peripheral blood from a larger panel of 40 patients (including the above mentioned 18 patients) was also collected, and serum was obtained by centrifugation. Concentrations of Syndecan‐1, TNF‐a, IL‐1β were determined by sandwich‐type enzyme‐linked immunosorbent assay (ELISA). Informed consent was obtained from all patients. The study was conducted according to the principles expressed in the Declaration of Helsinki and prior approval was obtained from the Medical Ethics Committee of Tianjin Medical University Cancer Institute and Hospital.

**Table 1:** Patient characteristics

Characteristics	Values
Age (years)	57 (range 30‐67)
Karnofsky performance score≧70	18 (100%)
Gender(female/male)	18/0 (100%/0)
Treatment	Radical external pelvic irradiation
Prior chemotherapy or surgery	
Yes	0
None	18 (100%)
FIGO Stage	
II	10 (55.55%)
III‐IV	8 (44.45%)
Squamous carcinoma	18 (100%)
Differentiation	
Well/Moderate	12 (66.67%)
Poor	6 (33.33%)
Diameter of tumour	
≥4 cm	7 (38.89%)
<4 cm	11 (61.11%)
Vaginal infiltration	
Presented	6 (33.33%)
None	12 (66.67%)
Lymph node metastasis	
Presented	13 (72.22%)
None	5 (27.78%)
Distant metastasis	
Presented	2 (11.11%)
None	16 (88.89%)

### 2.2 DNA extraction and 16S rRNA gene sequencing

Microbial metagenomic DNA was extracted using QIAamp DNA Micro Kit. Polymerase chain reactions were carried out with Phusion® High‐Fidelity PCR Master Mix. Bar‐coded primers targeting V4 region of 16S rRNA gene were 5’‐GTGCCAGCMGCCGCGGTAA‐3’ (515F) and 5’‐GGACTACHVGGGTWTCTAAT‐3’ (806R). The amplified products were purified with Qiagen Gel Extraction Kit. Sequencing was performed using a 250‐bp paired‐end sequencing protocol on an Illumina HiSeq2500 platform.

### 2.3 In vitro studies

The human normal colonic epithelial cell line consisting of FHC (foetal colon) was provided by Dr Liang Peng (The First Affiliated Hospital of Guangzhou Medical University), and cultured routinely in RPMI1640 medium. Faecal bacterial suspension was obtained as previously described.^13^ 1 × 10^5 ^FHC cells were seeded on the apical side of transwell filters (6.5 mm diameter inserts, 3.0 mm pore size) and reached confluence for 21 days to form maximal barrier function. Bacterial suspension was adjusted to reach an optical density of 0.05 (A_λ600_) and added to the apical chamber. Co‐cultures were incubated at 37°C for 5 h under microaerophilic conditions. Foetal colon cells were lysed in RIPA buffer. Proteins were detected by Western blot. Cytokine expression was detected by quantitative real‐time PCR. Integrity of cell monolayers was determined with transepithelial electrical resistance (TEER) using an epithelial tissue ohmmeter. Permeability of cell monolayers was evaluated with FITC‐dextran (4 kD, 1 mg/mL) flux from the apical to basolateral sides of the transwell filter by spectrophotometry at an excitation wavelength of 498 nm and an emission wavelength of 540 nm.^14^

### 2.4 Bioinformatics and statistical analysis

Paired‐end reads were assigned to samples based on their unique barcode and bioinformatic processing was done as previously described.^15^, ^16^ Briefly, quality control was performed using QIIME V1.7.0 software package to obtain high‐quality clean tags. The clean tags were compared with the reference database (Gold database, http://drive5.com/uchime/uchime_download.html) using UCHIME algorithm (UCHIME Algorithm, http://www.drive5.com/usearch/manual/uchime_algo.html) to detect chimera sequences and obtain effective tags. Sequences analysis was performed with Uparse software (V7.0.1001, http://drive5.com/uparse/). Operational taxonomic units (OTUs) clustering was done at 97% similarity level against the GreenGene Database (http://greengenes.lbl.gov/cgi-bin/nph-index.cgi) based on RDP classifier (V2.2, http://sourceforge.net/projects/rdp-classifier/) algorithm. At last, multiple sequence alignment was conducted using the MUSCLE software (V3.8.31, http://www.drive5.com/muscle/) to study phylogenetic relationship of different OTUs and the difference of the dominant species in different samples. α‐diversity was calculated by Simpson and Shannon indices.^17^ β‐diversity was analyzed by weighted and unweighted Unifrac distance.^18^ Statistical analysis was performed with *t* test, non‐parametric Mann‐Whitney, MetaStat, LEfSe, MRPP and ADONIS, etc.

For in vitro studies, data from three or more independent experiments was expressed as mean ± SE and processed using SPSS18.0 statistical software. Statistical analysis was performed with *t* test, non‐parametric Mann‐Whitney and factorial analysis. *P* < 0.05 from two‐sided tests was taken as statistical significance.

## 3 RESULTS

**Figure 1:**
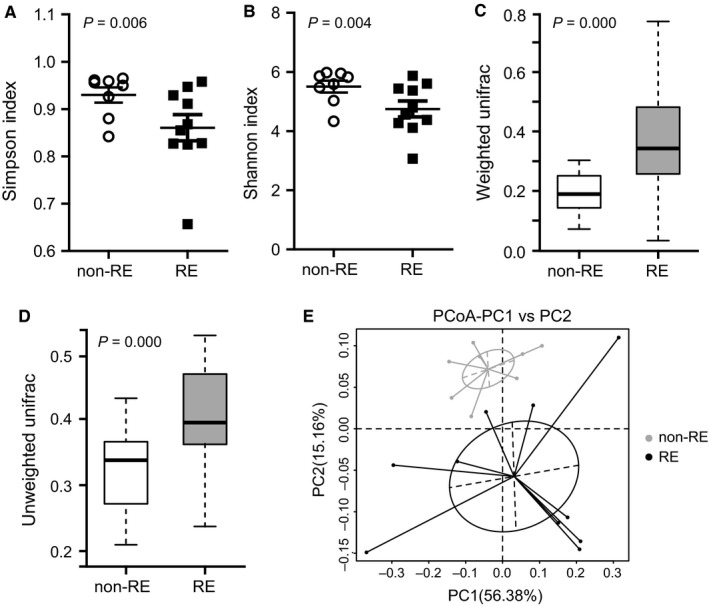
Analysis of diversity in cervical cancer patient with RE compared with patients without RE. α‐diversity was determined by (A) Simpson index and (B) Shannon index. β‐diversity was determined by (C) weighted and (D) unweighted Unifrac analysis of the distance matrix. (E) PCoA analysis based on weighted Unifrac distance matrices. Each sphere represents one sample. Samples separate into two clusters. non‐RE, cervical cancer patient without RE; RE, cervical cancer patient with RE

In total, we generated 1 617 140 paired‐end reads of high‐quality sequences (average 57 755 per sample). The total number of OTUs was 14 832 at 97% similarity level. Species accumulation boxplot showed that the gene richness approached saturation as a function of sample size, indicating the number of samples was sufficient to resolve most of the genera present (Figure S1). Then we investigated the richness and evenness of gut microbiota in patients with RE (RE group, N = 10) compared with patientswho did not have RE (non‐RE group, N = 8). α‐diversity of RE patients was markedly reduced as indicated by Simpson and Shannon indices (*ρ* = 0.006 and 0.004 respectively, Figure 1A,B). Similarity of microbiome community structures was further compared by Unifrac analysis of distance matrix with 10 000 permutations (Figure S2). Boxplot indicated a significantly higher β‐diversity of RE patients (*ρ* = 0.000, Figure 1C,D). We observed reduced α‐diversity but increased β‐diversity, indicating a less complex and more heterogeneous community in RE‐associated gut microbiota. PCoA analysis showed that RE cohort and non‐RE cohort separated substantially (Figure 1E). MRPP (*A* = 0.051, observed‐delta = 0.574, expected‐delta = 0.605, *ρ* = 0.015) and ADONIS (*R* ^2^ = 0.876, *ρ* = 0.013) analysis based on Bray‐Curtis distance further demonstrated that gut microbiota of RE group was distinct from the non‐RE group. The significant difference in clustering was also supported by AMOVA (analysis of molecular variance) analysis based on Unifrac distance (*ρ* = 0.040).

**Figure 2:**
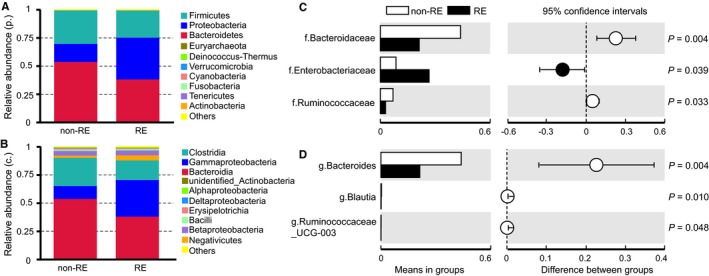
Alterations in the composition of gut microbiota in cervical cancer patient with RE compared with patients without RE. The relative abundance of bacteria in the faecal samples from the RE and non‐RE patients at the phylum (A) and class levels (B). Boxplots representing the average proportion of each 16S sequence read attributed to each taxon in patients on the family (C) and genus (D) levels. *White* non‐RE, samples of patients without RE, *Black* RE, samples of patients with RE

Then we compared the differences in taxonomic abundances between individuals with and without RE at various levels. At the phylum level, *Bacteroidetes* was the most predominant gut microbiota, contributing 38.59% and 54.12% in RE group and in non‐RE group respectively, followed by *Proteobacteria *(37.10% and 15.97%) and *Firmicutes* (24.01% and 29.66%) (Figure 2A). The relative abundance of *Proteobacteria* in RE patients was significantly higher (*ρ* = 0.028), while less abundance of *Bacteroidetes* and *Firmicutes* did not reach statistical significance. At the class level, a significant increase was observed in the abundance of *Gammaproteobacteria *in the RE group (32.41% vs 11.46%, *ρ* = 0.039, Figure 2B). Upon closer examination of taxonomic data, we noted that RE group was enriched with order *Enterobacteriales *(26.63% vs 8.55%, *ρ* = 0.038) and *Oceanospirillales *(0.059% vs 0.095%, *ρ* = 0.039) from the *Gammaproteobacteria *class. At the family level, eight families were presented at significantly altered proportions (Figure 2C, *ρ* < 0.05) in RE patients compared to non‐REs. Five families were increased including *Enterobacteriaceae* (*ρ* = 0.039), *Phyllobacteriaceae* (*ρ* < 0.001) and *Beijerinckiaceae* (*ρ* < 0.001), whereas *Bacteroidaceae* (*ρ* = 0.004) and *Ruminococcaceae* (*ρ* = 0.033) were decreased. Genus‐level analysis was more informative (Figure 2D). In RE patients, genus *Serratia, Bacteroides* and *Prevotella_9* were the most abundant, while the proportionate representation of *Bacteroides* was markedly reduced (21.23% vs 43.83%, *ρ* = 0.004). Other minor genera significantly less‐abundant in RE patients were *Blautia *(*ρ* = 0.010) and *Ruminococcaceae_UCG‐003* (*ρ* = 0.048).

**Figure 3:**
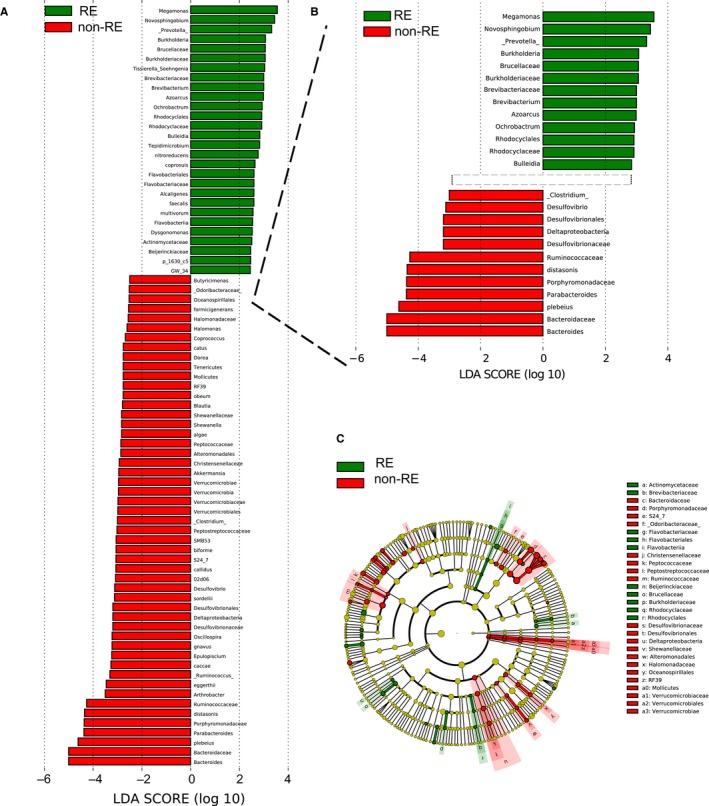
Taxonomic differences between patient with RE and patients without RE. (A, B) Linear discriminative analysis (LDA) effect size (LEfSe) analysis. (C) Cladogram showing differentially abundant taxonomic clades with an LDA score >4.0. *red* non‐RE, *green* RE

To identify the specific bacterial taxa associated with RE, we compared the composition of faecal microbiota using linear discriminant analysis effect size (LEfSe). It revealed 79 discriminative features (LDA score > 4, Figure 3A,B). Members of *Bacteroides, Bacteroidaceae* and *Plebeius* were enriched in non‐REs, whereas *Megamonas,* *Novosphingobium* and *Prevotella* were enriched in RE samples. The latter three could thus be used as biomarkers to identify RE patients. A cladogram represented the structure of faecal microbiota and the predominant bacteria was shown in Figure 3C. It provided the relationship between taxa at different taxonomic levels. For example, *Desulfovibrionaceae* (family) was under *Desulfovibrionales* (order) which was under *Deltaproteobacteris* (class).

**Figure 4:**
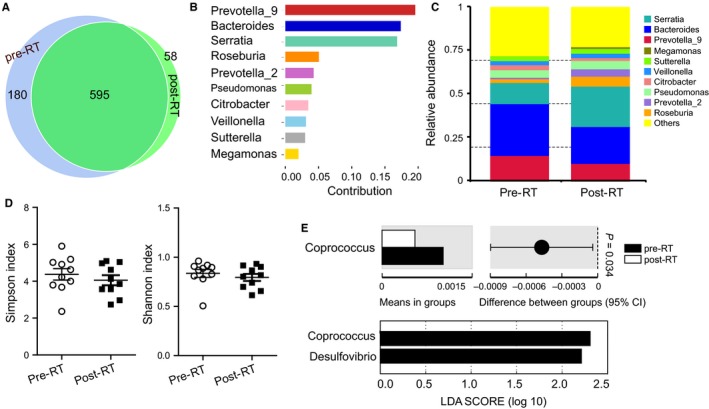
Faecal microbiota is associated with possibility of RE development. (A) Venn diagram representing the core operational taxonomic units (OTUs) in faecal samples obtained prior to and post radiotherapy in RE patients. (B) Average proportions of 16S sequence reads representing the core taxa. (C) Average relative abundance of 16S sequence reads representing core taxa. (D) Differences in α‐diversity as indicated by Simpson and Shannon indices. (E) Genus *Coprococcus *was enriched in pre‐RT patients. LEfSe analysis between pre‐RT patients and post‐RT patients. Pre‐RT, samples collected prior to radiotherapy from patients who later suffered RE; post‐RT, samples collected post‐radiotherapy from patients who were suffering RE

We also investigated microbial differences between faecal samples obtained prior to (pre‐RT condition) and post‐radiotherapy (post‐RT condition) in all the RE patients to identify association of any microbial profiles with the risk of developing RE. The results showed that 595 distinct OTUs were shared by all the RE patients over irradiation, 180 distinguished pre‐RT patients and 58 distinguished post‐RT patients (Figure 4A). The core set was characterized by genera *Prevotella_9, Bacteroides, Serratia, Roseburia, Prevotella_2, Pseudomonas, Citrobacter, Veillonella, Sutterella *and *Megamonas*. Most of these genera were differentially distributed (Figure 4B,C). Diversity (as measured with Simpson and Shannon indices) was decreased in patients who later suffered RE over radiotherapy, although no significant difference was observed (Figure 4D). Genus *Coprococcus *was obviously significantly enriched in pre‐RT samples (*ρ* = 0.034, Figure 4E‐upper). LEfSe analysis further revealed two discriminative features (LDA score > 4, Figure 4E‐lower); members of *Coprococcus *and *Desulfovibrio* were enriched in the pre‐RT samples. Therefore they might be used as biomarkers to identify patients most likely to develop RE.

**Figure 5:**
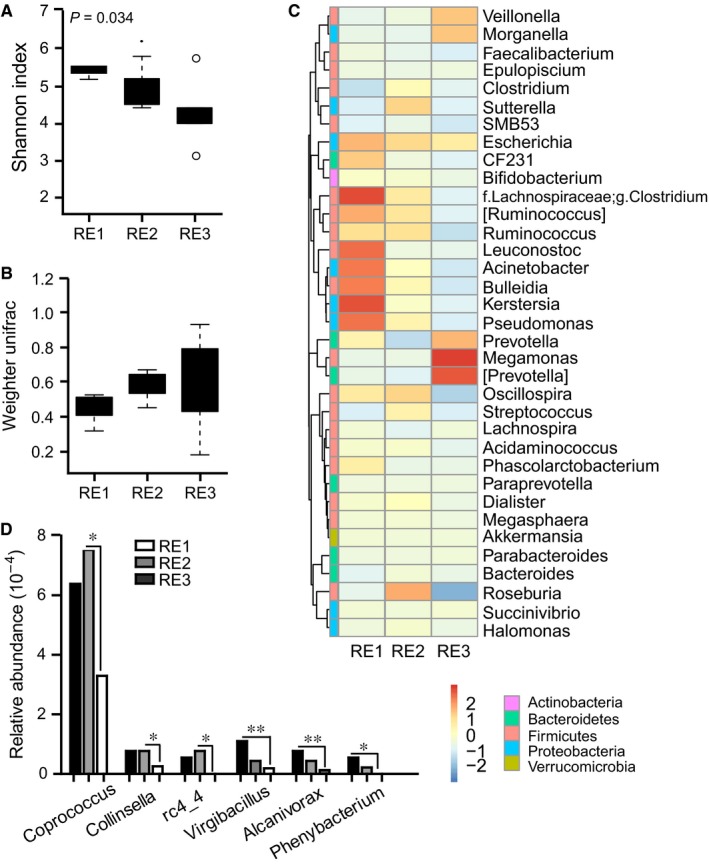
Faecal microbiota is associated with disease severity of RE. (A) Differences in α‐diversity in patients with mild to severe RE as indicated by Shannon index. (B) Differences in β‐diversity as indicated by weighted Unifrac analysis of the distance matrix. (C) Heatmap showing abundance distribution of the OTUs identified as key variables among patients with mild to severe RE. (D) Metastat analysis showing the relative abundances of the significant six bacteria at the genus level in patients with mild to severe RE

Patient samples were further classified into three groups according to the grade of RE using the Radiation Therapy Oncology Group (RTOG) grading system.^19^ Three patients in the RE group developed grade 1 radiation toxicity (RE1), three developed grade 2 toxicity (RE2) and another four developed grade 3 toxicity (RE3). No grade 4 or 5 was recorded. Relative to those with mild toxicity, patients with severe enteritis had a significantly reduced α‐diversity (*ρ* = 0.034, Figure 5A) but a non‐significantly increased β‐diversity (Figure 5B). Patients at grade 3 RE had lowest α‐diversity and highest β‐diversity among all the RE patients, indicating a potential trend of gradual microbial response to radiation inflammation. Analysis of microbial composition revealed a grade‐related microbial feature (Figure 5C). Proportionally, six bacterial taxa were enriched in RE1, and two in RE3. Metastat analysis showed that among the mild‐grade RE‐enriched genus, three were significantly more abundant in RE1 patients, including *Virgibacillus* (*ρ* = 0.008)*, Alcanivorax *(*ρ* = 0.010) and *Phenybacterium *(*ρ* = 0.038); three were more abundant in RE2 patients, including *Coprococcus *(*ρ* = 0.044)*, Collinsella *(*ρ* = 0.022)*, *and *rc4_4* (*ρ* = 0.020) (Figure 5D).

**Figure 6:**
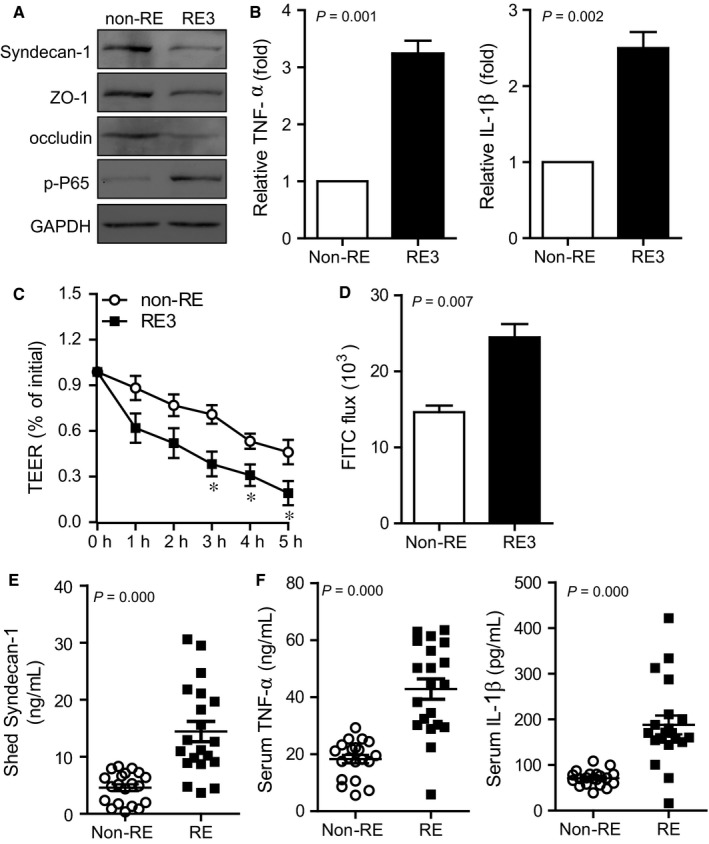
Faecal microbiota is associated with intestinal inflammation and barrier function. Foetal colon cells were co‐cultured with irradiated microbiota from patients with grade 3 RE. (A) Barrier‐associated proteins and NF‐κB activity were determined using Western blot. (B) Cytokine secretion was determined using quantitative PCR. (C) Epithelial integrity was determined with TEER. (D) Epithelial permeability was determined with FITC‐dextran flux. Patients’ serum were collected. Levels of shed Syndecan‐1 (E) and cytokines TNF‐α and IL‐1β (F) were detected by ELISA

An epithelial monolayer cell co‐culture model was used to explore the effects of radiation‐induced microbial dysbiosis on epithelial inflammatory response. Following co‐culture with bacterial suspensions from RE3 patients, compared with suspension from non‐RE patients, inflammatory and barrier markers were significantly down‐regulated. Membrane Syndecan‐1 was released from the cell surface. Tight junction protein ZO‐1 and occludin were decreased. P65, a canonical component of NF‐κB pathway, was phosphorylated as well (Figure 6A). RE‐derived microbiota also stimulated remarkable TNF‐α and IL‐1β secretion (*ρ* = 0.001 and 0.002, Figure 6B). Transepithelial electrical resistance was decreased consistently and significantly in a time‐dependent manner through cells co‐incubated with RE3‐derived microbiota (*ρ* < 0.05, Figure 6C). Consistent with the higher TEER drop, higher FITC‐dextran permeation was also observed (*ρ* = 0.007, Figure 6D). Furthermore, soluble Syndecan‐1, as well as cytokines TNF‐α and IL‐1β in the serum of RE patients were all significantly higher than in patients without RE (*ρ* < 0.001, Figure 6E, F).

## 4 DISCUSSION

Since its first report in 1897,^20^ the incidence of RE continues to rise tremendously in recent years. The occurrence of symptoms like diarrhoea, rectal bleeding and tenesmus impair patients’ quality of life, increase healthcare costs and often lead to suspension of the treatment. Why some patients develop severe RE while others do not is a long‐standing and ‐perplexing question. In this study, we for the first time identified alterations in gut microbial profiles following RE development using high‐throughput 16S rRNA gene sequencing based on the Illumina HiSeq platform. Within limitation of the small patient cohort, we demonstrate that (a) patients with different grades and phases of RE have their own characteristic gut microbiota, and (b) the radiation‐induced dysbiosis in turn promotes inflammatory responses in the host.

Several previous studies have indicated that patients receiving radiotherapy exhibit marked changes in gut microbiota, some of which were specific to particular patients. For example, the *Firmicutes/Bacteroidetes* ratio was markedly altered prior to radiotherapy in patients who later developed diarrhoea.^21^ Patients with acute post‐radiotherapy diarrhoea had profound increase in *Actinobacteria* and *Bacilli*, and decrease in *Clostridia*.^22^ In our study, unique microbial signatures were observed at the tested grades and phases of RE. Patients with RE had a significantly altered microbial diversity and composition over irradiation. Genus *Serratia, Bacteroides* and *Prevotella_9* were more abundant in RE patients, while *Bacteroides* was markedly reduced. Furthermore, we found a RE grade‐related microbial signature. There was a lowest α‐diversity while a highest β‐diversity of gut flora in grade 3 RE patients, as well as significantly different abundance of several selected genera between patients with severe or mild enteritis. More striking findings were associated with changes in gut microbiota before radiation. Patients who later progressed to RE had obviously enriched *Coprococcus* before irradiation and decreased α‐diversity after irradiation. These results may be related to differences in the severity of local mucosal inflammation, or changes in epithelial permeability or barrier. Factors which were not investigated here might also play a part. However, these results have strongly suggested the importance of gut microbiota and the need for further and more detailed investigation.

We did some preliminary in vitro experiments to explore whether and how the microbiota affected the radiation‐associated tissue damage. Incubating colonic epithelial cells with faecal bacteria from patients with severe RE impaired cell layer integrity, increased cell layer permeability and stimulated cytokine secretion and NF‐κB activation. Thus the dysbiotic microbiota might in part directly induce barrier dysfunction and inflammatory response on the epithelial cells. Otherwise, microbiota significantly differed following local radiation treatment. And these changes paralleled with the cytokine profile in patients with or without RE. It was suggested the host immune response upon irradiation shapes the microbial community structure. When cells are exposed to radiation, leucocytes infiltrate into the irradiated normal cells. Various signalling pathways are activated, accompanied with secretion of pro‐inflammatory cytokines, shedding of mucosa, disruption of barrier and initiation of coagulation cascade.^23^ For example, pathway analysis on the gene expression profiles has identified radiation‐induced time‐, dose‐ and even segment‐dependent up‐regulation of TNF‐α, claudin‐2, MMP7 and EDA2R.^24^ Radiation also provokes increase in MPO activity and CXC chemokine levels.^25^ Activation of these pathways suggest that colon sustains severe mucosal inflammation and barrier disruption, and might influence and disturb the balance of microecology. We previously demonstrated that loss of Syndecan‐1 in the inflamed intestine impaired normal intestinal barrier and led to bacterial translocation through mucosa.^14^ Winter et al^26^, ^27^ found that host‐derived nitrate in response to mucosal inflammation conferred a growth advantage to commensal *Escherichia coli* or pathogenic *Salmonella enterica *in the mice intestine. Taken together, although it is tricky to decipher the question of cause and effect, these data are still sufficient to confirm that the unique radiation‐induced dysbiosis is closely associated with inflammatory response.

Our results suggested that the pre‐existing changes in gut microbial ecology may serve as a predictive marker to identify patients who are more likely to progress to RE during pelvic irradiation, in agreement with Wang et al's proposal.^21^ Moreover, our data suggested possibility to prevent or treat RE by targeting the gut microbiota. In mouse model, gavage of *Lactobacillus rhamnosus GG* before radiation repositioned COX‐2 expression through TLR‐2/MyD88 signalling and reduced epithelial apoptosis and crypt loss from radiation injury.^28^ In patients, prevention of radiotherapy‐induced mucositis by probiotics has been investigated in several clinical trials.^29^, ^30^ Although the results were inconsistent, and strong evidence is lacking, there was still some promising data. Chitapanarux's randomized study included 63 patients treated with pelvic radiotherapy concurrent with weekly cisplatin chemotherapy. As compared with placebo, treatment with live *Lactobacillus acidophilus *plus *Bifidobacterium bifidum* resulted in improved stool consistency and less usage of anti‐diarrhoeal medication.^31^ In Urbancsek's larger randomized trial with 206 irradiated patients, supplementation with *Lactobacillus rhamnosus* led to less frequently needed anti‐diarrhoeal drugs.^32^ Furthermore, L. Fuccio^33^ systemically reviewed clinical trials including the above two. However, no significant differences were confirmed between probiotic supplementation and placebos. Despite the few available trials and the presence of significant clinical and statistical heterogeneity might limit the analysis, encouraging results have been indeed observed in some patients. Because not all probiotics exert favourable effects, possibly owing to variability of probiotics and patient characteristics, the importance of identifying the classification of patients and the ideal type and dose of bacterial strains need to be addressed in further high‐quality clinical trials. Moreover, cancer patients are generally at risk of disease‐ or treatment‐related immunosuppression. Microbial preparation may induce detrimental effects in these individuals; note some published reports of septic complications because of probiotics.^34^ Therefore, safety concerns about the use of probiotics should also be carefully investigated.

In conclusion, we reported the comprehensive analysis of gut microbiota in patients with RE using faecal samples by high‐throughput 16S rRNA sequencing. We identified the radiation‐induced impaired gut microbiota and its relationship with RE. Our results will be helpful for the prediction and treatment of cancer patients receiving pelvic irradiation and suffering from RE. Furthermore, multicenter, randomized and placebo‐controlled trials are needed to confirm this.

## CONFLICT OF INTEREST

The authors declare that there are no conflicts of interest.

## AUTHORS’ CONTRIBUTIONS

ZQW and ZYY designed the study. ZQW, QXW, XW performed the actual laboratory analyses. QXW, LZ, JC and BLZ obtained the samples and analysed the data. ZQW, WW, YC and ZYY wrote and revised the manuscript.
