# Supplementary figures and images for: Gut microbial dysbiosis is associated with development and progression of radiation enteritis during pelvic radiotherapy
Source: J Cell Mol Med. 2019 Mar 25;23(5):3747–56. doi: 10.1111/jcmm.14289 (PMC6484301; doi:10.1111/jcmm.14289)

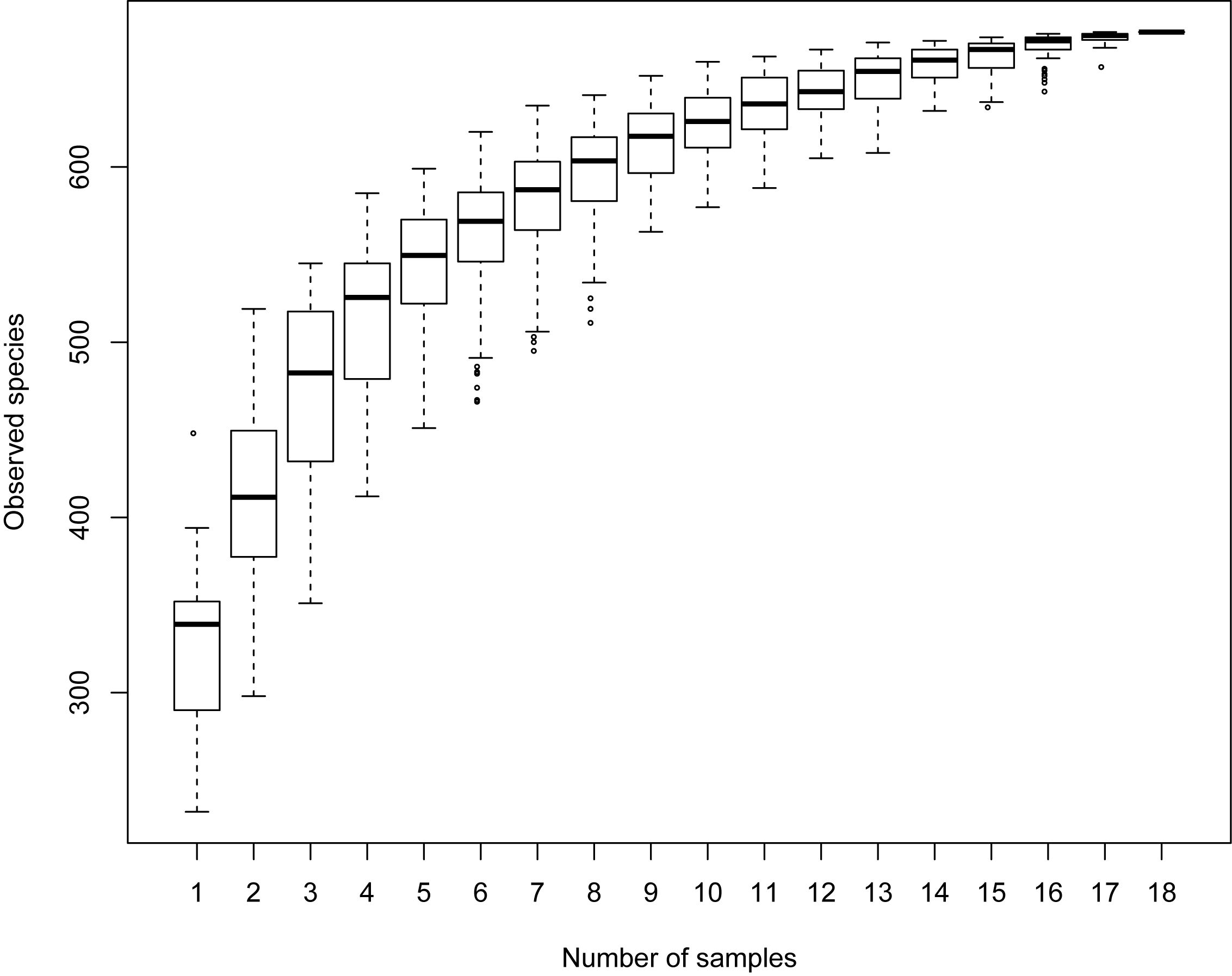

Supplement: Supplementary file 1 [file JCMM-23-3747-s001.tif]

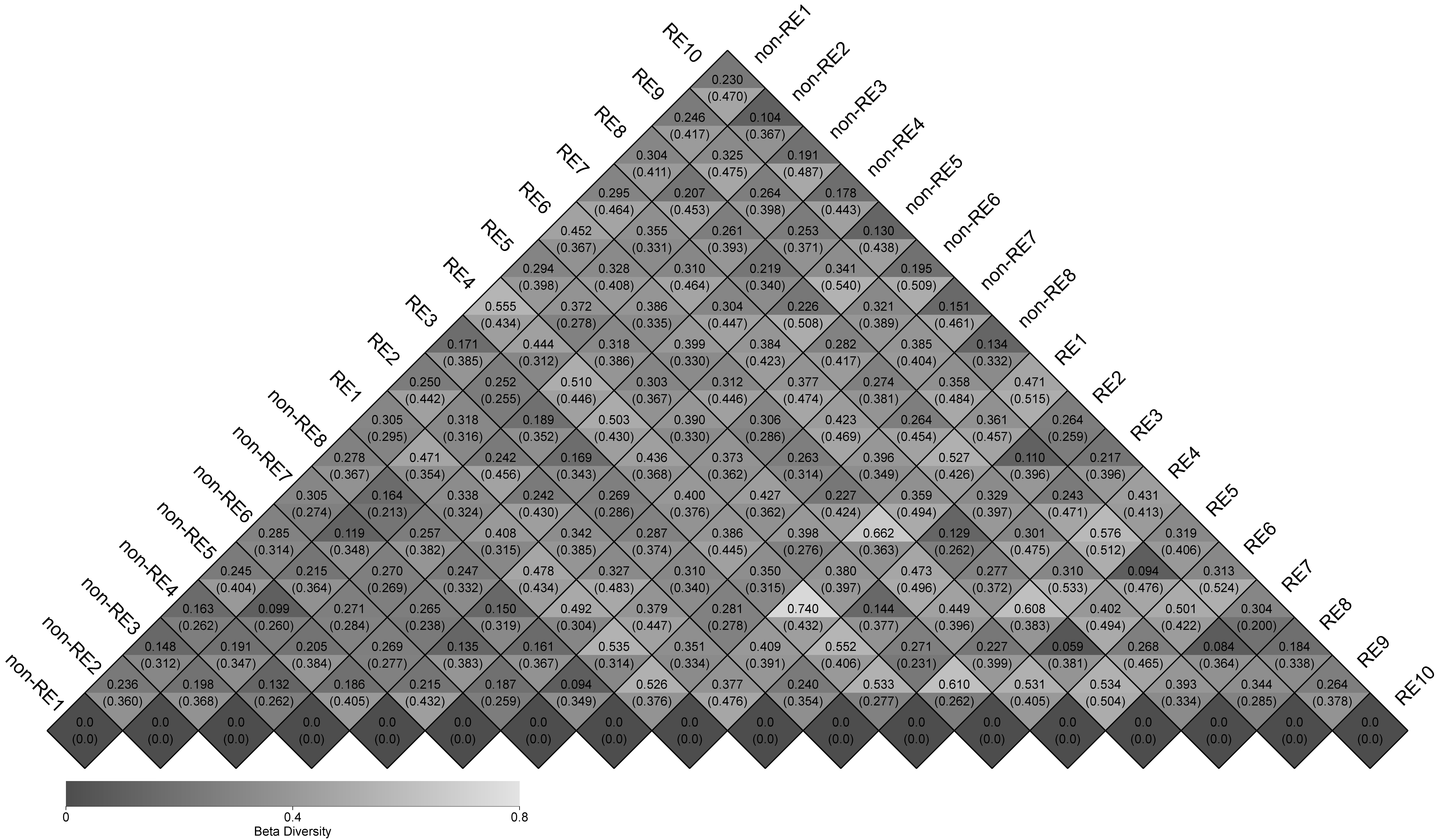

Supplement: Supplementary file 2 [file JCMM-23-3747-s002.tif]
